# The *Arabidopsis* NPF3 protein is a GA transporter

**DOI:** 10.1038/ncomms11486

**Published:** 2016-05-03

**Authors:** Iris Tal, Yi Zhang, Morten Egevang Jørgensen, Odelia Pisanty, Inês C. R. Barbosa, Melina Zourelidou, Thomas Regnault, Christoph Crocoll, Carl Erik Olsen, Roy Weinstain, Claus Schwechheimer, Barbara Ann Halkier, Hussam Hassan Nour-Eldin, Mark Estelle, Eilon Shani

**Affiliations:** 1 Department of Molecular Biology and Ecology of Plants, Tel Aviv University, Tel Aviv 69978, Israel; 2 Howard Hughes Medical Institute and Section of Cell and Developmental Biology, University of California San Diego, La Jolla, California 92093, USA; 3 DynaMo Center, Faculty of Science, University of Copenhagen, Thorvaldsensvej 40, 1871 Frederiksberg C, Denmark; 4 Plant Systems Biology, Technische Universität München, 85354 Freising, Germany; 5 Department of Plant and Environmental Sciences, Faculty of Science, University of Copenhagen, Thorvaldsensvej 40, 1871 Frederiksberg C, Denmark

## Abstract

Gibberellins (GAs) are plant hormones that promote a wide range of developmental processes. While GA signalling is well understood, little is known about how GA is transported or how GA distribution is regulated. Here we utilize fluorescently labelled GAs (GA-Fl) to screen for *Arabidopsis* mutants deficient in GA transport. We show that the NPF3 transporter efficiently transports GA across cell membranes *in vitro* and GA-Fl *in vivo*. *NPF3* is expressed in root endodermis and repressed by GA. NPF3 is targeted to the plasma membrane and subject to rapid BFA-dependent recycling. We show that abscisic acid (ABA), an antagonist of GA, is also transported by NPF3 *in vitro.* ABA promotes *NPF3* expression and GA-Fl uptake in plants. On the basis of these results, we propose that GA distribution and activity in *Arabidopsis* is partly regulated by NPF3 acting as an influx carrier and that GA–ABA interaction may occur at the level of transport.

## Abstract (web summary)

Transport of the plant hormone gibberellin is required for normal plant growth and development. Here, Tal *et al*. show that NPF3 is able to transport gibberellin *in vitro*, and provide evidence that it is required for normal gibberellin distribution and activity in plants.

## Graphical abstract

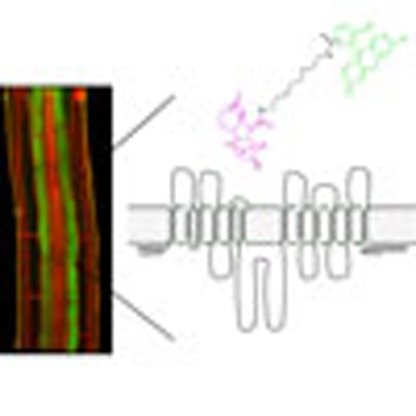

Unlike animals, plants are sessile organisms that must integrate biotic and abiotic stimuli with their genetic programme to grow and reproduce in a dynamic environment. Growth and development are regulated in response to environmental inputs such as temperature, nutrient status, water, pathogens and light. To enable signal integration, plants employ hormone systems that are exquisitely controlled at multiple levels such as hormone biosynthesis, metabolism, perception and signalling^1^,^2^,^3^. In addition, the distribution and cellular concentration of plant hormones are regulated, thereby enabling highly coordinated cellular responses^4^,^5^,^6^,^7^,^8^,^9^. For example, the combined activities of auxin influx and efflux carrier proteins generate local hormone maxima, minima and auxin gradients that are required for various developmental processes^10^,^11^. The recent identification of abscisic acid (ABA), strigolactone and cytokinin transporters^6^,^7^,^8^,^9^,^12^ implies that, like auxin, the *in planta* distribution of these and possibly also other hormones is determined by transporters.

Gibberellins (GAs) are tetracyclic diterpenoid hormones that control many developmental processes such as seed germination, root and shoot elongation, flowering and fruit patterning. The genetic or agrochemical manipulation of GA biosynthesis or signalling is a widespread practice in agriculture. The best-known examples are the dwarfing alleles of wheat *REDUCED HEIGHT*1 and rice *SEMIDWARF*1 that are one of the foundations of the so-called ‘Green Revolution' and resulted in enormous increases in global crop yields after World War II^13^. Whereas research over the past 15 years has elucidated the GA biosynthetic and signalling pathways at the molecular level, it remains to be determined precisely where GAs are active within the plant and how GAs are transported from sites of synthesis to sites of action. Studies using radiolabelled GA and grafting experiments with GA mutants, have demonstrated that GAs are mobile hormones and that their movement is essential for proper plant growth^14^,^15^,^16^,^17^,^18^,^19^. A recent *Arabidopsis* grafting experiment with a variety of GA biosynthetic mutants demonstrated long-distance transport of the inactive GA_12_ (ref. 20). Many GAs are membrane permeable weak acids that can accumulate in the cytoplasm due to an ion-trapping mechanism^21^. To explain how GAs can pass through the plasma membrane as a part of cell-to-cell transport, the existence of active GA carriers has been proposed^22^.

We have recently explored the *in planta* distribution of several bioactive GAs in *Arabidopsis thaliana* roots using custom-made fluorescein-tagged (fluorescent) GA molecules (GA-Fls)^23^. Interestingly, these biologically active GA-Fls specifically accumulated in the endodermal cells of the root elongation zone but not in the meristematic or differentiation zones, whereas free fluorescein was detected in all tissues^23^. The data suggest that the specific accumulation of GA-Fl in the endodermis is tightly regulated^23^,^24^. However, the identity of GA transporters involved in this process has remained elusive.

Here we describe a search for GA transporters by screening for mutants defective in GA-Fl accumulation. We show that mutants lacking *NPF3*, a poorly characterized member of the NPF family, are deficient in the uptake of fluorescent GA into elongating root endodermal cells. *NPF3* overexpression causes a striking accumulation of GA-Fl in all root cells and dramatic inhibition of root and shoot growth. Experiments in *Xenopus* oocytes show that NPF3 is an active GA importer. We also demonstrate that NPF3 transports ABA in oocytes, suggesting that ABA and GA transport may be linked through the dual specificity of several NPF transporters.

## Results

### NPF3 transports GA-Fl into elongating root endodermal cells

**Figure 1:**
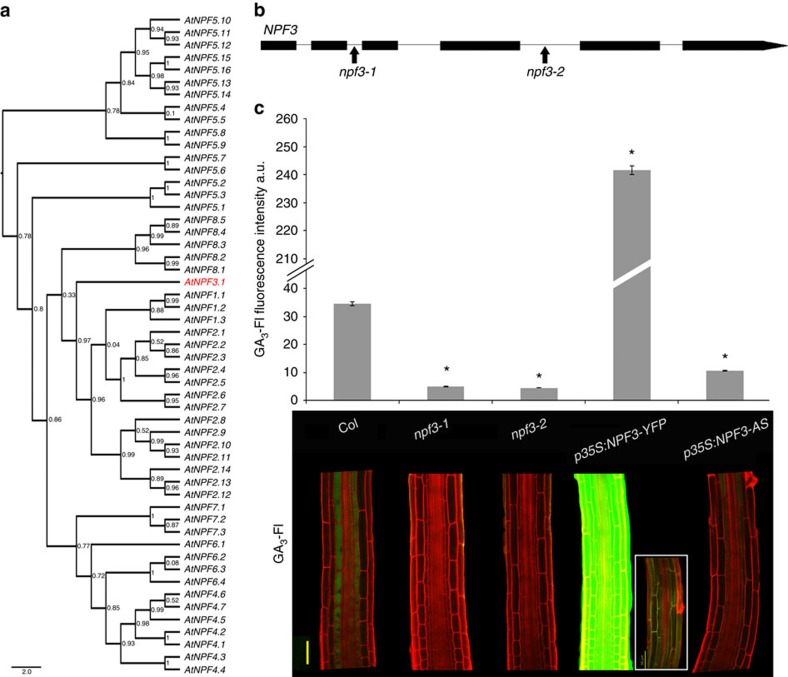
*NPF3* is required for GA-Fl accumulation in the elongating endodermal cells of the root. (**a**) Phylogenetic tree of the *Arabidopsis NPF* (*NRT/PTR*) family. *NPF3.1* (*NPF3*, At1g68570) shown in red. (**b**) *NPF3* (At1g68570) gene model. Arrows indicate the positions of the T-DNA insertion for *npf3-1* (SALK_130095) and *npf3-2* (GK-356G08). (**c**) Quantification and distribution of fluorescently tagged GA_3_ (GA_3_-Fl) in elongating endodermal cells of roots (5 μM GA_3_-Fl, 3 h treatment). Top; quantification of fluorescence intensity averages±s.e. (4 roots imaged per genotype, 17 sampling points per root; *n=68*). * Significantly different relative to Col at *P*≤0.001 by Student's *t*-test. Bottom, confocal images of representative roots. Box shows untreated *p35S:NPF3-YFP* root using the same imaging setup. AS, antisense. Bar, 50 μm.

To identify putative GA transporters, we screened a collection of ≈180 transfer DNA (T-DNA) insertion mutants of the ATP-binding cassette and the NITRATE TRANSPORTER1/PEPTIDE TRANSPORTER (NPF) transporter family members for seedlings that were defective in GA-Fl accumulation in the root endodermis^25^,^26^ (Supplementary Data 1). Members of both transporter families have been shown to function as plant hormone transporters^6^,^8^,^9^,^12^,^27^,^28^,^29^,^30^. The screen was performed visually by testing 10 seedlings per T-DNA line using the confocal microscope. The screen identified a T-DNA line that was deficient in the accumulation of GA-Fl. The T-DNA insertion disrupted *NPF3* (*At1g68570, NPF3.1*), a poorly characterized member of the *NPF* gene family (Fig. 1a)^31^,^32^. Two independent insertion mutants, *npf3-1* (SALK_130095) and *npf3-2* (GK-356G08) (Fig. 1b), accumulated very low levels of GA_3_-Fl and GA_4_-Fl in the elongating endodermal cells compared with the wild-type (WT) but accumulated normal levels of free Fl (Fig. 1c, Supplementary Fig. 1a). Reverse transcription–PCR (RT–PCR) experiments indicated that some regions of the *NPF3* transcript accumulated to high levels in the T-DNA lines (Supplementary Fig. 1b,d–e); however, neither allele generated full-length *NPF3* RNA (Supplementary Fig. 1b–c). Both T-DNA lines were backcrossed to Col-0. Genetic analysis revealed that the uptake defect segregated as a single recessive mutation completely linked to the insert (*n*=100). These results suggest that *NPF3* may be involved in GA transport into the elongating endodermal cells of the root.

*NPF3* belongs to an evolutionarily conserved but strongly expanded and diversified family of transporters with 53 members in *Arabidopsis*. *Arabidopsis NPF1.1* and *NPF4.6/AIT1* transport auxin and ABA, respectively^12^,^30^. Recent work showed that various NPF proteins can transport GA, ABA and jasmonic acid-isoleucine (JA-Ile)^33^,^34^. NPF3 is conserved in all plant lineages and its orthologs share a high degree of identity^26^, suggesting a fundamental physiological role. Since loss of *NPF3* function abolished GA_3_-Fl accumulation in the elongating endodermal cells we hypothesized that misexpressing *NPF3* in additional cell types would affect GA_3_-Fl accumulation in the root. We introduced a construct into Col-0 plants in which NPF3 was fused to yellow fluorescent protein (YFP) and placed under the control of the *35S* promoter (*p35S:NPF3-YFP)*. *NPF3* RNA levels were 16- and 5-fold higher (depending on the line) in *p35S:NPF3-YFP* plants compared with WT plants (Supplementary Fig. 1d). Indeed, accumulation of GA_3_-Fl was markedly enhanced in these lines (Fig. 1c). This result strongly supports the hypothesis that NPF3 facilitates GA transport *in planta*. Overexpression of the *NPF3* gene in reverse orientation (*p35S:NPF3*-antisense) resulted in two and threefold reduction (depending on the line) in *NPF3* transcript levels compared with WT (Supplementary Fig. 1d) and in reduced GA_3_-Fl accumulation in elongating endodermis (Fig. 1c). Overall, the results indicate that *NPF3* is required for accumulation of GA_3_-Fl in endodermal cells in the root elongation zone.

### NPF3 localization and regulation

**Figure 2:**
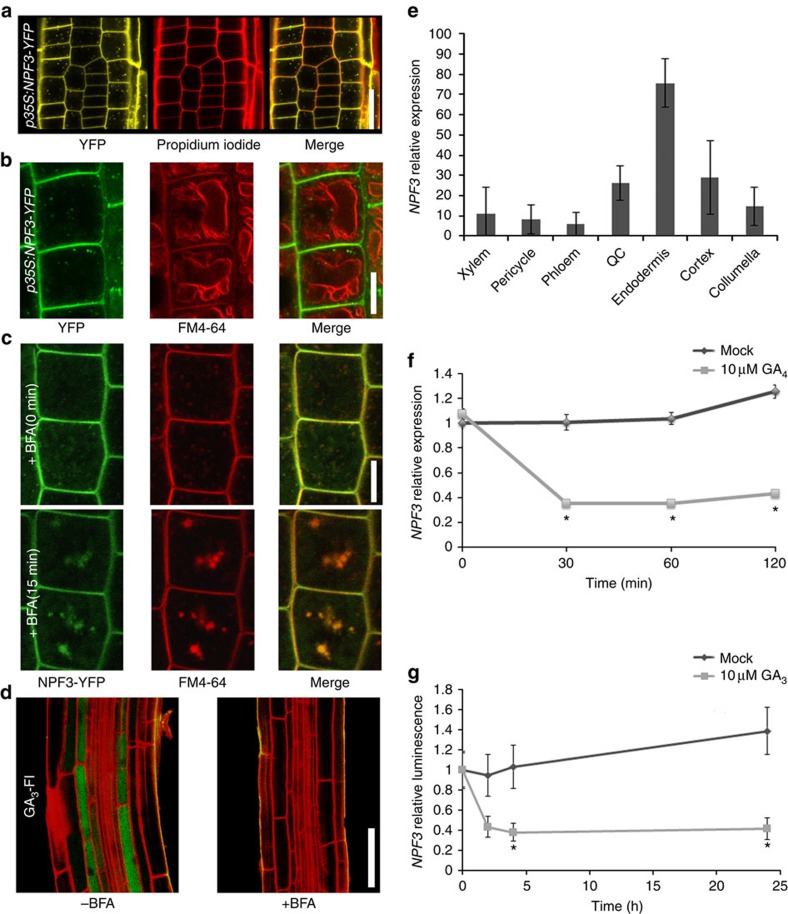
NPF3 is targeted to the plasma membrane in a BFA-dependent manner and is transcriptionally repressed by GA. (**a**) Root confocal microscopy images of NPF3-YFP localization counterstained with Propidium Iodide. Bar, 20 μm. (**b**) Confocal microscopy images of root epidermis cells from 7-day-old *p35S:NPF3-YFP* seedlings. Vacuolar tonoplast was pulse-stained for 15 min with FM4-64 (2 μM), transferred to dye free liquid media for 60 min, and imaged subsequently. Bar, 10 μm. (**c**) *p35S:NPF3-YFP* plants treated for 0 and 15 min with the trafficking inhibitor Brefeldin A (BFA) (50 μM). Bar, 10 μm. (**d**) Confocal images of WT roots immersed in GA_3_-Fl (5 μM 3 h treatment) with and without BFA treatment (50 μM 14 h treatment). Bar, 50 μm. (**e**) *NPF3* expression levels derived from root cell type-specific protoplasts *Brady et al*.^40^. (**f**) *NPF3* relative expression in response to 10 μM GA_4_ treatment. Seedlings were pretreated with paclo (5 μM, 6 days). RNA levels were quantified by qRT–PCR with *ACTIN8* as a reference gene. Values are mean±s.d. (*n*=3). (**g**) Luminescence intensity of *pNPF3:LUC* seedlings treated with mock or 10 μM GA_3_ for 0, 2, 4 and 24 h. Values are mean±s.e. (*n*=24). * Significantly different relative to respective mock at *P*≤0.001 by Student's *t*-test.

Our results suggest that NPF3 is a GA transporter. Indeed, *in silico* analysis predicts a putative 12 transmembrane domain carrier protein (Supplementary Fig. 2a). Plant hormone transporters localize to the plasma membrane^7^,^8^,^9^,^29^,^35^,^36^, as well as intracellular compartments such as the endoplasmic reticulum and vacuole^37^,^38^,^39^. To address NPF3 subcellular localization, we utilized our *p35S:NPF3-YFP* lines. Confocal microscopy of *p35S:NPF3-YFP* root tips showed that NPF3 localizes to the plasma membrane and intracellular membrane compartments (Fig. 2a). By imaging NPF3-YFP in root tip cells where the vacuoles are small and by using chemical and genetic markers that stain the tonoplast and plasma membrane, we were able to rule out vacuolar localization (Fig. 2b and Supplementary Fig. 2b). These results seemingly contradict previous experiments involving transient expression in tobacco that suggested localization of NPF3 orthologs from cucumber (*CsNitr1-L, CsNPF3.2)* to chloroplasts^32^. Moreover, *in silico* analysis showed that the N terminus of *Arabidopsis* NPF3 lacks a chloroplast targeting sequence that is predicted for *CsNitr1-L*^31^. To further investigate NPF3 trafficking to the plasma membrane, we treated *p35S:NPF3-YFP* seedlings with Brefeldin A (BFA), an inhibitor of ARF–GTP exchange factors, for 15 min and imaged NPF3-YFP by confocal microscopy. This treatment resulted in the depletion of NPF3-YFP from the plasma membrane and accumulation of the transporter in intracellular vesicles, indicating that NPF3 is rapidly targeted to the plasma membrane in a BFA-dependent manner (Fig. 2c). Consistent with this, we observed that BFA-treated roots are deficient in GA_3_-Fl uptake (Fig. 2d). Thus, NPF3 localization on the plasma membrane is required for GA_3_-Fl accumulation in the elongating endodermal cells. Co-treatments of *p35S:NPF3-YFP* seedlings with GA or paclobutrazol (paclo; an inhibitor of GA synthesis) with and without BFA did not show a significant difference compared with respective control treatments, implying that NPF3 subcellular localization or its BFA sensitivity are not directly affected by GA_4_ levels (Supplementary Fig. 2c-d).

According to published root cell-specific transcriptomic data^40^ and other publicly available microarray data, *NPF3* is expressed in roots, albeit at very low levels. Interestingly, a high-resolution root spatiotemporal microarray expression map shows *NPF3* transcripts at higher levels in the root endodermis compared with other tissues in roots (Fig. 2e)^40^. Moreover, additional support for endodermis localization of NPF3 was obtained by analysing cell type-specific expression data (Supplementary Fig. 3a) derived from microarray studies of RNA bound to ribosomes, which were immunoprecipitated by the use of epitope-tagged ribosomal protein from seedlings^41^. In combination, the localization of *NPF3* transcripts and translation are consistent with a role for NPF3 in GA-Fl accumulation in the root endodermis.

Several plant hormone transporters are regulated at the transcriptional level by their hormone substrate^8^,^37^,^42^. To evaluate the response of *NPF3* to GA, we analysed *NPF3* transcript abundance following GA treatment. Ten-day-old Col-0 seedlings pretreated with paclo show strongly reduced *NPF3* RNA levels in response to 10 μM GA_4_ treatment as measured by quantitative PCR experiments (Fig. 2f). In agreement, *pNPF3:LUC* (luciferase) 10-day-old seedlings treated with 10 μM GA_3_ for 1–24 h showed reduced LUC signal (Fig. 2g). These results indicate that high levels of GA repress *NPF3* expression and thus inhibit GA uptake. Published data show that *NPF3* is induced by ABA (10 μM, 3 h) and salt stress (150 mM, 12 h)^43^,^44^,^45^. These results were reproduced for ABA in our quantitative PCR experiments (Supplementary Fig. 4a). Accordingly, we show that GA_3_-Fl accumulation in the endodermis is induced by ABA and NaCl treatments in a concentration-dependent manner (Supplementary Fig. 4b). The ABA induced GA_3_-Fl uptake into the elongating endodermal cells does not take place in *npf3-1* mutant, and therefore is NPF3 dependent (Supplementary Fig. 4b).

### *NPF3* loss and gain-of-function growth phenotypes

**Figure 3:**
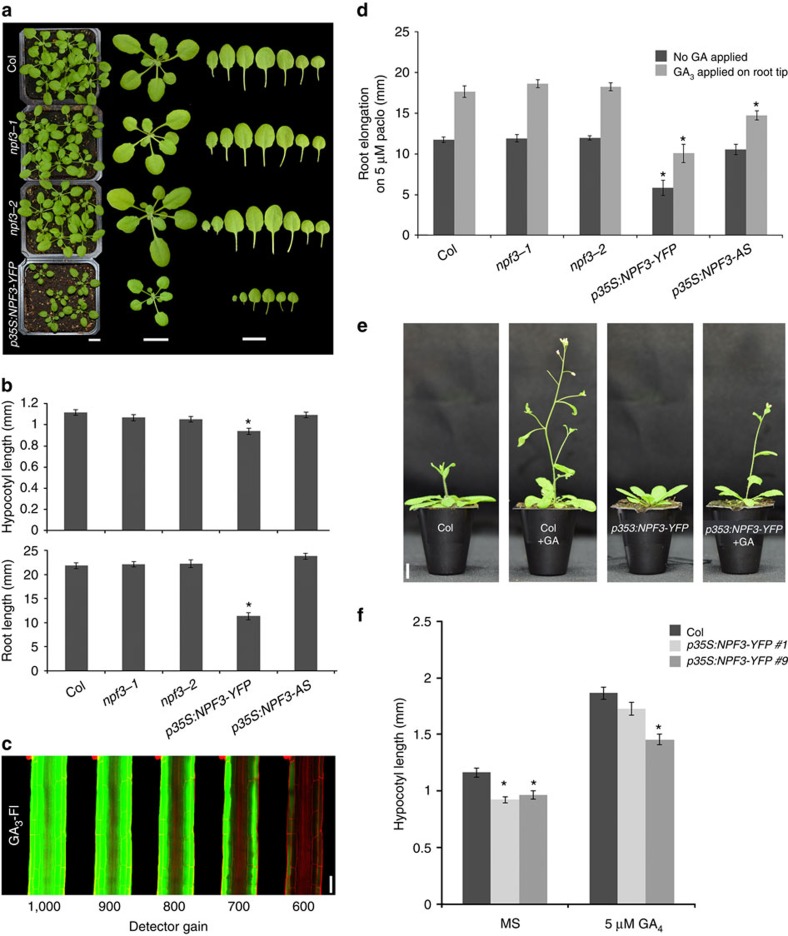
Overexpression of *NPF3* mimics GA deficiency. (**a**) Phenotypes of 3 week-old *npf3* mutants and *p35S:NPF3-YFP* lines compared with WT (Col-0). Bar, 1 cm. (**b**) Quantification of root and hypocotyl length of 7-day-old seedlings growing on MS media. Values are means±s.e. of 11 seedlings per genotype in two independent experiments *n=*22. (**c**) GA_3_-Fl accumulation in *p35S:NPF3-YFP* plants under reduced detector gains, (5 μM GA_3_-Fl, 1.5 h treatment). Bar, 50 μm. (**d**) Graph presenting root elongation on 5 μM paclo, and response to exogenously applied 5 μM GA_3_. Shown are means±s.e. of at least 11 seedlings per genotype. The experiment was repeated three times. (**e**) Representative images of 4 week-old WT and *p35S:NPF3-YFP* plants grown on soil with or without 10 μM GA_3_ spraying, *n=*12, bar, 1 cm. (**f**) Hypocotyl length of 2 independent *p35S:NPF3-YFP* lines and WT (Col-0) seedlings germinated and grown on MS with or without 5 μM GA_4_. Values are means±s.e. of 13 seedlings per genotype. * Significantly different relative to respective WT at *P*≤0.001 by Student *t*-test.

**Figure 4:**
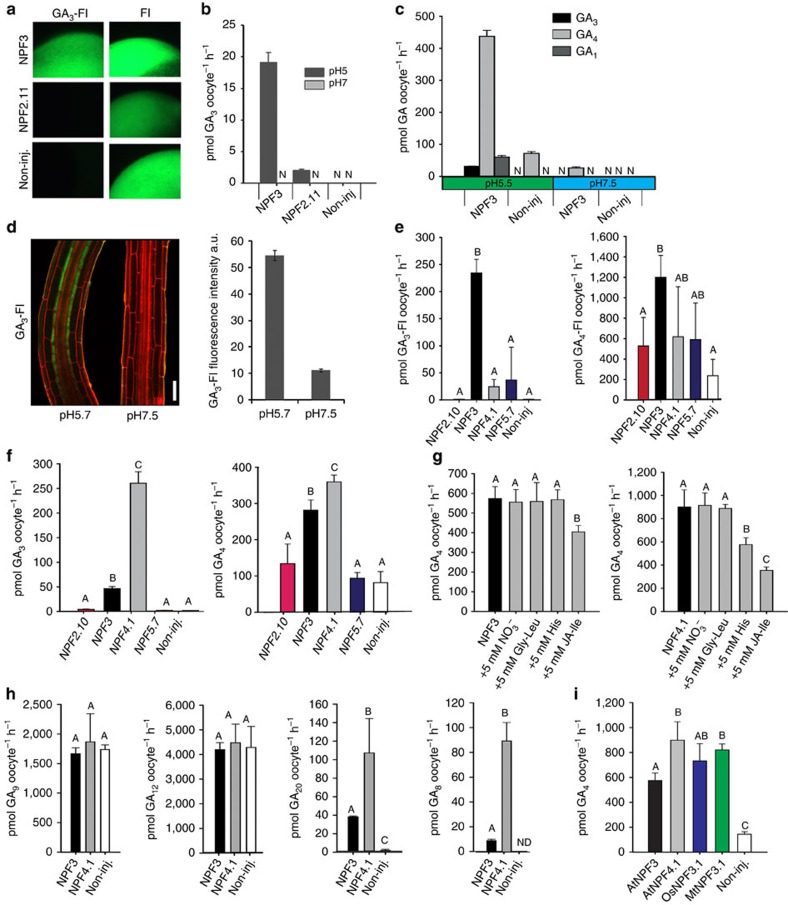
NPF3 is a GA transporter in *Xenopus laevis* oocytes. (**a**) Uptake of GA_3_-Fl and fluorescein by NPF3, NPF2.11 (GTR2) and non-injected oocytes. Oocytes were incubated for 30 min in 100 μM GA_3_-Fl (pH5). Picture shows a representative oocyte from 3 oocytes. (**b**) Uptake of GA_3_ by NPF3, NPF2.11 (GTR2) and non-injected oocytes at pH5 and pH7. Oocytes were incubated for 1 h in 100 μM GA_3_ (pH5 or pH7) (*n*=3). N: below detection limit. (**c**) Bioactive GA_1_, GA_3_ and GA_4_ uptake at pH 5.5 and pH 7.5 by *NPF3*. *NPF3*-expressing or non-injected oocytes were incubated for 1 h in 100 μM with the indicated GAs (*n*=3). N: below detection limit. (**d**) Confocal images of WT root elongation zone immersed in GA_3_-Fl (5 μM, 3 h) in indicated pHs. Right graph: quantification of GA_3_-Fl fluorescence intensity. Presented are averages±s.e. roots per genotype and 26 sampling points per root (*n*=104). Bar, 50 μm. (**e**) Uptake of GA_3_-Fl and GA_4_-FI by oocytes expressing the indicated endodermis expressed NPFs (*n*=4). (**f**) Uptake of GA_3_ and GA_4_ by oocytes expressing the indicated endodermis expressed NPFs (*n*=4). (**g**) Competition of GA_4_ uptake mediated by NPF3 and NPF4.1 (AIT3) expressing oocytes. Oocytes expressing either NPF3 or NPF4.1 were exposed to 300 μM GA_4_ alone or 300 μM GA_4_ together with either 5 mM NO_3_^−^, 5 mM dipeptide (Gly-Leu), 5 mM histidine (His) or 5 mM JA-isoleucine (JA-Ile; *n*=5). (**h**) Uptake of indicated GA precursors and catabolite mediated by NPF3 and NPF4.1 (AIT3) expressing and non-injected oocytes. Oocytes were incubated for 1 h in 100 μM of the indicated GA (at pH5) (*n*=5). (**i**) Uptake of GA_4_ by *Oryza sativa* and *Medicago truncatula* NPF3.1 orthologs as defined by ref. 26. Oocytes expressing *A. thaliana* NPF3 (AtNPF3.1), *O. sativa* NPF3.1 (OsNPF3.1) or *M. truncatula* NPF3.1 (MtNPF3.1) or non-injected oocytes were incubated for 1 h in 300 μM GA_4_ at pH5 (*n*=5). In all graphs: Error bars are s.d. unless indicated otherwise. Groups are determined by one-way analysis of variance and (*P*<0.05). non-inj., non-injected.

The fluorescent GA experiments suggested that NPF3 is involved in bioactive GA transport in the root. To study *NPF3* regulation in GA-mediated plant growth and development, we compared WT, *NPF3* loss of function and overexpression lines (*p35S:NPF3-YFP)*, assessing GA-mediated traits such as germination, root and shoot elongation and flowering time. Both *npf3-1* and *npf3-2* mutants lines were like the WT with respect to all these traits (Fig. 3a,b) despite their failure to accumulate the fluorescently tagged hormone. The lack of a mutant phenotype in the *npf3* mutants despite the strong effect on GA-Fl uptake is puzzling. It is possible that there are alternative functionally redundant GA transporters that compensate in the absence of *NPF3*. This hypothesis is supported by functional transport experiments described below (Fig. 4e,f, Supplementary Fig. 6).

In contrast, *p35S:NPF3-YFP* plants exhibit delayed germination, decreased hypocotyl growth, and a strong reduction in root and shoot growth (Fig. 3a,b, Supplementary Fig. 5). These defects are similar to those seen in GA-deficient plants^46^,^47^. The growth inhibition caused by *NPF3* overexpression might be caused by the retention of GA at sites of synthesis, thus preventing movement of GA to key growth sites. If this is correct, one might expect stronger accumulation of GA-Fl in the outer layers (GA-Fl is applied exogenously) for *p35S:NPF3-YFP* roots. Indeed, GA_3_-Fl accumulated at higher levels in the epidermal layer compared with the inner layers (Fig. 3c), suggesting that the epidermal layer has imported GA_3_-Fl and trapped the molecule in the cell in a *NPF3*-dependent manner. In addition, expression of the *GA2ox1* gene, encoding a GA-inactivating enzyme, was transiently reduced in response to GA compared with WT (Supplementary Fig. 5c). The striking ectopic GA-Fl accumulation in additional cells of *p35S:NPF3-YFP* seedlings, accompanied by GA-related developmental defects observed in these lines, support a role for *NPF3* in GA responses.

To further understand the role of *NPF3* in GA-mediated root growth, we examined the response of *NPF3* loss- and gain-of-function lines to paclo and applied GA. Paclo is an inhibitor of GA synthesis that causes reduced root growth that can be rescued by applying exogenous bioactive GA. The *npf3* mutants responded to paclo and applied GA like WT. In comparison, *p35S:NPF3-YFP* and *p35S:NPF3*-antisense plants showed a reduced growth response to GA. (Fig. 3d). The response of *p35S:NPF3-YFP* seedlings to GA is consistent with the hypothesis that ectopic expression of *NPF3* inhibits bioactive GA movement to key growth sites. *p35S:NPF3*-antisense lines showed reduced *NPF3* expression (Supplementary Fig. 1d), while no additional change in expression level could be detected in other family members that were shown to transport GA in yeast assays^33^, and are expressed in the root tip (Supplementary Figs 5d, 6b).

Long-term treatment of *p35S:NPF3-YFP* lines with GA might compensate for the altered distribution of endogenous hormone. In agreement, long-term (10 and 21 days) GA treatment of the *p35S:NPF3-YFP* line promoted shoot growth and flowering, largely rescuing the hypocotyl elongation, (depending on *NPF3* overexpression strong/weak line; Fig. 3e,f), while partially rescuing the root growth phenotype (once a day for 4 days, 1 μl application, 5 μM GA_3_ to root tip, in paclo background; Fig. 3d). To investigate whether NPF3 promotes GA activity as opposed to GA storage and degradation, *p35S:NPF3-YFP* seedlings were grown on increasing concentrations of GA. While WT plants showed reduced growth and yellow shoot colour on high GA concentration (50 μM), *p35S:NPF3-YFP* showed similar phenotypes when grown on 15 μM GA (Supplementary Fig. 5e). This result suggests that NPF3 promotes accumulation of GA in GA responding cells and that *NPF3* is involved in GA-mediated plant growth and development.

Recent work showed that various NPF proteins can transport ABA and nitrate^26^. To test the role of NPF3 in ABA and nitrate response, we tested *NPF3* overexpression lines, T-DNA mutants and antisense lines on ABA and nitrate assays. We could not detect any difference to the WT in response to nitrate for any of the tested lines (Supplementary Fig. 5g–h). However, overexpression of *NPF3* resulted in a significant reduction in germination with and without ABA treatment (Supplementary Fig. 5i). Interestingly, we observed a small but significant decrease in germination in the *npf3-2* mutant allele and *NPF3* antisense line at high ABA concentrations (3 μM; Supplementary Fig. 5i). *NPF3* overexpression but not *npf3* loss-of-function lines showed significant root response to ABA compared with WT (Supplementary Fig. 5j). It is, therefore, likely that the strong *NPF3* overexpression phenotype is driven by the mislocalization of both hormones. These results together with the results showing that ABA induces *NPF3* expression and GA-Fl accumulation in the elongating endodermal cells, suggest that NPF3 may be involved in ABA localization and response.

### NPF3 is a GA transporter

Our results indicate that NPF3 transports GA-Fl *in planta*. To elucidate the mechanisms underlying GA transport by NPF3, we used the *Xenopus* oocyte expression system. Uptake experiments showed that NPF3 mediated GA_3_-Fl uptake into oocytes (Fig. 4a), confirming that NPF3 is a GA-Fl importer. In contrast, the glucosinolate transporter 2 (NPF2.11/GTR2) and non-expressing control oocytes did not import GA_3_-Fl into oocytes (Fig. 4a). Oocytes expressing NPF3 exposed to 100 μM GA_3_ at pH 5 (∼apoplastic pH conditions) showed a 19.1-fold increase in GA_3_ uptake compared with non-injected oocytes, and 10-fold higher than GTR2 uptake (Fig. 4b), indicating that NPF3 can transport native GA. To determine whether NPF3 transports other active GAs present in plants, we exposed NPF3-expressing oocytes to 100 μM GA_1_, GA_3_ or GA_4_ at pH 5.5 and 7.5. Oocytes expressing NPF3 accumulated ∼14 fold higher GA_4_ levels compared to GA_3_, and ∼7 fold higher levels compared to GA_1_ at pH 5.5 (Fig. 4c). This indicates that GA_4_, which is the active endogenous GA in *Arabidopsis* is the preferred GA substrate of NPF3 (Fig. 4c). Most NPF importers characterized to date are proton symporters that utilize the inwardly directed electrochemical proton gradient (ΔμH^+^) between the plant apoplast (∼pH 5) and cytoplasm (∼pH 7.5) to drive import of substrates^48^. Oocytes expressing NPF3 showed an increased uptake of GA_4_, GA_3_ and GA_1_ at pH 5.5 compared with pH 7.5 (Fig. 4c). This indicates that transport by NPF3 is dependent on the pH gradient. In agreement, seedlings exposed to pH 7.5 media showed reduced accumulation of GA_3_-Fl in the root elongation zone compared with roots growing in pH 5.7 media (Fig. 4d,e).

We exposed NPF3-expressing oocytes to increasing concentrations of GA_4_ and calculated the GA_4_ transported by NPF3 into the oocytes following 60 min incubation by subtracting uptake in non-injected oocytes (diffusion). This yielded a saturation curve from which we estimate an apparent *K*_m_ of NPF3 towards GA_4_ of 0.5 mM (Supplementary Fig. 6a). Kinetic uptake assays of weak acids are, however, notoriously difficult to interpret. The propensity of weak acids to diffuse across cellular membranes at acidic pH and the cumulative nature of the uptake assays makes it difficult to accurately estimate the apparent *K*_m_. As an example, we previously estimated the apparent *K*_m_ value for glucosinolate uptake by GTR2 using liquid chromatography mass spectrometry (LC-MS) and electrophysiology-based uptake assays. LC-MS-based uptake assays estimated a *K*_m_ value that was approximately fivefold higher (unpublished data) compared with the more accurate value determined by electrophysiology^49^. We thus judge that the *K*_m_ of NPF3 towards GA_4_ is likely to be an overestimation.

It was recently reported that a large number of NPF transporters promote GA uptake in yeast when utilizing a GA-dependent yeast two-hybrid protein–protein interaction approach^33^. Besides NPF3, six of the apparent GA-importing *NPF* genes were expressed in different tissues in the root tip. These include *NPF2.3*, *NPF2.10/GTR1*, *NPF4.1/AIT3*, *NPF4.2/AIT4*, *NPF5.6* and *NPF5.7* (Supplementary Fig. 6b). Using the *Xenopus* oocyte system, we compared the ability of NPF3 and the three transporters NPF2.10, NPF4.1 and NPF5.7 to import GA_3_-Fl and GA_4_-FI. Interestingly, NPF3 displayed transport activity towards GA_3_-Fl and GA_4_-Fl whereas the other genes imported neither of the labelled GA species significantly above levels detected in control oocytes (Fig. 4e). We then tested the ability of the four transporters to import non-conjugated GA_3_ and GA_4_. In contrast to the qualitative evidence for GA import provided previously^33^, only NPF3 and NPF4.1 imported GA_3_ and GA_4_ into the oocytes to levels significantly above those found in control oocytes (Fig. 4f). This indicates that the number of putative GA transporters suggested previously^33^,^34^ may be an overestimation. Interestingly, NPF4.1 imported GA_3_, (exogenous to *Arabidopsis*) to fivefold higher levels compared with NPF3, whereas import of GA_4_ (endogenous to *Arabidopsis*) was similar between the two proteins (Fig. 4f). Taken together, these results highlight that before identification and characterization of the transporters, caution must be taken when using fluorescent labelling to follow phytohormone mobility *in planta*. In this study, application of GA-Fl helped us identify NPF3 as a putative GA transporter but also indicated that NPF3 was solely responsible for GA accumulation in root endodermis. In fact, biochemical characterization shows that NPF4.1 likely acts redundantly and may compensate for *NPF3* loss of function *in planta*.

As the NPF family of transporters possesses a remarkably wide substrate specificity^26^, we performed competition experiments using known substrates of the family. GA_4_ import by NPF3 and NPF4.1 was competed with 15-fold excess concentration of NO_3_^−^ (ref. 50), dipeptide (Gly-Leu)^51^, histidine^52^ or JA-Ile^33^. NPF3-mediated transport of GA_4_ was not significantly affected by excess nitrate, dipeptide nor histidine and was weakly affected by JA-Ile. In comparison, NPF4.1-mediated uptake of GA_4_ was weakly affected by excess histidine whereas JA-Ile strongly inhibited GA_4_ uptake (Fig. 4g, Supplementary Fig. 6c–d). These observations suggest that NPF3 is predominantly a GA transporter. In comparison, our and previous studies suggest that NPF4.1 is a multi-specific transporter capable of importing several structurally unrelated phytohormones including ABA, GA and JA-Ile.

Recent work has identified the GA-intermediate GA_12_ as a long-distance transported form of inactive GA in *Arabidopsis*^20^ and the GA-intermediate GA_20_ as a transported form in *Pisum sativum*^53^. Consequently, we tested whether NPF3 and NPF4.1 transported selected commercially available intermediates (GA_9_, GA_12_ and GA_20_) and the catabolite GA_8_ of the GA biosynthetic pathway. At pH5, GA_9_ and GA_12_ precursors of GA_4_ accumulated to very high levels in oocytes and to the same extent in NPF3- and NPF4.1-expressing and non-injected oocytes due to diffusion (Fig. 4h). We were therefore not able to determine whether NPF3 and NPF4.1 transport these two intermediates. In comparison, GA_20_ accumulated to detectable levels in oocytes expressing NPF3 and NPF4.1, but to barely detectable levels in non-injected oocytes (Fig. 4h). Similarly, GA_8_, catabolite of GA_1_, accumulated to detectable levels in oocytes expressing NPF3 and NPF4.1, but below detection levels in non-injected oocytes (Fig. 4h). This suggests that NPF3 is able to transport GA_20_ in *Arabidopsis.* Interestingly, transport of the GA_8_ catabolite by NPF3- and NPF4.1-expressing oocytes indicated that intercellular transport of GA catabolites may occur in *Arabidopsis*.

Phylogenetic analysis suggests that NPF3 is conserved in all plant lineages with orthologs sharing a high degree of identity^26^. We tested GA_4_ transport capability of the NPF3 orthologs in the grass *Oryza sativa* (OsNPF3.1) and the legume *Medicago truncatula* (MtNPF3.1)^26^. OsNPF3.1 and MtNPF3.1 imported GA_4_ into oocytes to similar levels as AtNPF3, which indicates functional conservation of GA transport activity in orthologous NPF3 transporters (Fig. 4i).

Finally, as several members of the NPF family have been shown to transport ABA and auxin^12^,^30^,^33^,^34^, we tested NPF3 transport activity towards these plant hormones at pH 5.5 and pH 7.5. NPF3 accumulated GA_4_ and ABA in oocytes to comparable levels (Supplementary Fig. 6f). Dual GA/ABA substrate specificity was also reported for NPF4.1, previously identified as an ABA transporter also permeable for GA_3_ (ref. 12). This suggests that ABA and GA transport may be linked through the dual specificity of several NPF transporters. Uptake of the auxin indole-3-acetic acid by NPF3 was similar to non-injected control oocytes (Supplementary Fig. 6e). Thus, it is possible that NPF3 in our experimental setup plays a particularly important role for GA-Fl uptake, but acts redundantly with at least, NPF4.1/AIT3 or other GA transporters with respect to transport of non-conjugated ABA and GA. Altogether, our results show that NPF3 is a GA transporter that is functionally conserved across dicot and monocots and that appears functionally redundant with additional GA transporters in the elongating root endodermis.

## Discussion

GAs are fundamental for many aspects of plant growth and development. Here we shed light on the cellular mechanisms regulating GA transport in the root by revealing that NPF3 is a GA importer. An important question that this study could not fully address is whether *NPF3* regulates GA cellular influx to promote GA activity or to inhibit GA response by GA degradation and/or storage. However, the fact that the endodermis is known to have an important role in GA response^54^,^55^, and *NPF3* overexpression results in hypersensitivity to high levels of GA suggests that *NPF3* is functioning to promote GA response. Although this study reveals that NPF3 can transport GA *in vitro*, and GA-Fl in the root, the complete GA transport mechanism is not clear. Since cytosolic pH 7.5 is driving GA de-protonation through the ion trap mechanism, the majority of GA_4_ is expected to be captured, with the limited ability to move from cell to cell^22^. The presence of GA exporters might explain how GA moves locally at the tissue or cellular level; however, these are yet to be discovered. It is possible that putative GA efflux carriers act redundantly and are therefore difficult to identify in genetic screens.

The NPF family of proton-coupled transporters is involved in nitrogen assimilation in eukaryotes and bacteria. In most plant species, NPF members have evolved to transport nitrate as well as additional specialized metabolites and hormones. This complexity makes it difficult to assess the biological role of the NPF proteins. How a transporter recognizes such different metabolites and hormones is an intriguing question. An interesting example of such diversity is the dual-affinity-specificity nitrate/auxin transporter *NRT1.1* (ref. 30). The dual sensor/transceptor model proposes that NO_3_^−^ sensing function of NRT1.1 is due to its dual NO_3_^−^/auxin transport activity and that the NO_3_^−^ signal transduced by NRT1.1 is an NO_3_^−^-dependent modification of auxin transport in lateral root development^30^. On top of the multisubstrate specificity, this and other recent studies suggest that several NPFs can transport GA^33^,^34^. These results present an additional level of regulation of the GA response. It is intriguing to consider how *NPF3* transports two antagonistic hormones, GA and ABA (possibly with different affinities), while *NPF3* is induced by ABA and repressed by GA. We hypothesize that differences in spatiotemporal expression pattern as well as differences in subcellular localization and transport affinity among the NPF transporters towards GAs might explain the complex specificity and redundancy of GA transport. We expect future studies to dissect the complex network of multisubstrate specificity versus functional redundancy towards the same substrate that have evolved in the NPF family.

## Methods

### Plant material and growth conditions

All *Arabidopsis thaliana* lines used in this work are in Col-0 background. T-DNA insertion mutants were obtained from the Arabidopsis Biological Resource Center and Nottingham Arabidopsis Stock Centre. Homozygous mutants were selected by PCR using primers listed in Supplementary Table 1.

Seeds were plated on medium containing 0.5 × Murashige-Skoog (MS) medium, 1% sucrose and 0.8% agar on vertical plates, stratified for 2 days at 4 °C then transferred to growth chambers (Percival CU41L5) at 21 °C, 100 μE m^−2^  S^−1^ light intensity under long day light (16 h light/8 h dark).

### Cloning of *NPF3* overexpression and reporter lines

*NPF3* was amplified with primer combinations listed in Supplementary Table 2, cloned into pENTR/D-TOPO (Invitrogen K2400) and subsequently cloned into binary destination vectors using LR Gateway reaction (Invitrogen 11791). We have examined the phenotype and YFP fluorescence for 13 independent *NPF3-YFP* lines (Supplementary Fig. 5f). The lines were partially silenced at the T_3_ generation. We continued with two lines that showed reduced silencing and high *NPF3* RNA level (line #1 and 9; Supplementary Figs 1d and 5). All experiments were carried out with *p35S:NPF3-YFP#9* unless otherwise indicated.

### Hormone and inhibitor application

Chemicals were supplied to the agar medium at concentrations as indicated for each experiment in figure legends (starting from 10 mM stock solutions). Seedlings were placed on agar plates and roots were uniformly supplemented with chemicals. GA-Fl (5 μM) was applied in liquid MS media. Time points and chemicals concentrations are indicated for each experiment in the figure legend.

### Imaging and analysis

Seedlings were stained in 10 mg l^−1^ propidium iodide (PI) for 1 min, rinsed and mounted in water. Seedlings were imaged on a laser scanning confocal microscope (Zeiss LSM 780 inverted microscope), with argon laser set at 488 nm for fluorescein excitation, 561 nm laser for PI excitation. Emission filters used were 493–548 nm filter for fluorescein derivatives, and 583–718 nm filter for PI emission. Image analysis and signal quantification were done with the measurement function of ZEN lite 2012 software. The number of quantified biological repeats and sampling points is indicated for each graph in figure legends.

### Statistical analysis

Two-tailed Student's *t*-test was performed whenever two groups were compared. Statistical significance was determined at *P*<0.001 unless otherwise indicated.

### Luciferase reporter assay

Transgenic lines expressing luciferase under *NPF3* promotor of 2Kb upstream of the ATG start codon, designated *pNPF3:LUC*, were grown for 10 days on MS horizontal plates and sprayed with solution containing 1 mM potassium luciferin (Promega E1601) and 2% Triton X-100. Plants were assayed for luciferase activity 14 h after spraying using BioSpace PhotonIMAGER. Plates were then flooded with liquid MS with or without 10 μM GA_3_ and assayed again at indicated times. Luminescence was quantified using M3Vision Software.

### Phenotype characterization

To assess germination, seeds were plated on horizontal MS plates, germination rate determined as seed coat rupture after 2 days scored under a Zeiss Stemi 2000-C stereo microscope.

For root and hypocotyl length measurements, seedlings were imaged using Zeiss Stemi 2000-C stereo microscope and measured using ImageJ software (http://rsbweb.nih.gov/ij/index.html).

To assess etiolation, seedlings of indicated lines were sown on sucrose free MS, exposed to 100 μE m^−2^ S^−1^ fluorescent light for 4 h, followed by 3 days at the dark. Hypocotyl length quantified as described above.

For root GA response assays, seeds were germinated on MS were transferred to 5 μM paclo after 4 days. On the next day, root length was marked. 1 μl of 5 μM GA_3_ diluted in water was applied to root tips for the three subsequent days. Roots were imaged and measured on day 10.

### Quantitative RT–PCR

For all experiments except results presented in Fig. 2f and Supplementary Fig. 5c, total RNA was isolated from the indicated plant materials using RNeasy Plant Mini Kit (QIAGN 74,904). DNA was removed by RQ1 RNase-free DNase (Promega M6101). Total RNA (2 μg) converted to complementary DNA (cDNA) using M-MLV Reverse Transcriptase (Promega M1701) with oligo(dT)15 primer according to manufacturer protocols. Quantitative RT–PCR was performed with 40 ng cDNA in a final volume of 10 μl with Fast SYBR Green Master Mix (ABI 4385612) using Step One Plus System and software (ABI). The reaction conditions included 40 amplification cycles, (3 s at 95 °C; 30 s at 60 °C). Three technical repeats were performed for each cDNA sample, and at least three biological repeats were used for each treatment. The relative quantification was calculated with the ΔΔCt method, *PP2A* used as reference gene. Primers are specified in Supplementary Table 3. For results presented in Fig. 2f and Supplementary Fig. 5c, plant materials were transferred to 5 μM paclo following germination on MS plates. After 6 additional days, seedlings were treated with 10 μM GA_4_ in liquid MS for the indicated time periods. Total RNA was isolated from plants using NucleoSpin RNA plant kit and DNA was removed by an on-column treatment with rDNase (Macherey-Nagel, Düren, Germany). 2 μg of total RNA was subsequently reverse transcribed with M-MuLV Reverse Transcriptase (Fermentas, St Leon-Rot, Germany) using an oligo(dT) primer. The cDNA equivalent of 30–50 ng of total RNA was used in a 10 μl PCR reaction in a CFX96 Real-Time System Cycler (BioRad, Freiburg, Germany) with SsoAdvanced Universal SYBR Green Supermix (Bio-Rad, München, Germany) with three technical replicates in a CFX96 Real-Time System Cycler (Bio-Rad) in a 40-cycle step amplification protocol (10 s at 95 °C; 25 s at 60 °C). The relative quantification was calculated with the ΔΔCt method and *ACT8* as a control. Relevant primers used are listed in Supplementary Table 3.

### NPF3 trafficking analysis

*p35S:NPF3-YFP* seedlings were grown for 5 days on MS plates, transferred to 10 μM paclo or mock plates for 2 additional days, then treated with 10 μM GA_4_ or mock for 2 h in liquid media. Before imaging, seedlings were stained with 2 μM FM4-64 (mock) or FM4-64 with BFA for 15 min.

### Gene synthesis and cloning for transport assays

The genes *NPF3*, *NPF4.1*, *NPF2.10*, *NPF2.11* and *NPF5.7* were part of a previously published transporter library^56^. *Os06g15370* (*OsNPF3.1*) and *Medtr5g055000* (*MtNPF3.1*) were synthesized by ThermoFisher Scientific Geneart with USER overhangs^56^,^57^. Coding sequences were cloned into the pNB1 oocyte expression vector^58^ that was made USER cloning compatible^56^,^57^ and verified by sequencing.

### Oocyte preparation and complementary RNA injection

*Xenopus* oocytes were purchased as defolliculated *Xenopus* oocytes (stages V–VI) from Ecocyte Biosciences (Germany). Injection of 50 nl complementary RNA (500 ng μl^−1^) into *Xenopus* oocytes was done using a Drummond NANOJECT II (Drummond Scientific). Oocytes were incubated for 3 days at 17 °C in Kulori (90 mM NaCl, 1 mM KCl, 1 mM MgCl_2_, 10 mM MES) pH 7.5 before assaying.

### Hormone uptake assays

GA_3_, indole-3-acetic acid, ABA, Gly-Leu, Histidine and GA_4_ were obtained from Sigma-Aldrich, GA_1_ was obtained from Toronto Research Chemicals, GA_3_-Fl and GA_4_-Fl were synthesized and characterized as previously described^23^. Briefly, N-Boc-2,2′-(ethylenedioxy) diethylamine was first reacted with 5-(and-6)-carboxyfluorescein succinimidyl ester to form a fluorescein-linker. Following deprotection, fluorescein-linker was conjugated to the C6 carboxylic acid of either GA_3_ or GA_4_ to form the corresponding GA_3_-Fl or GA_4_-Fl, respectively. GA_12_, GA_20_, GA_9_, GA_8_ and JA-Ile were obtained from OlChemIm Ltd. (Czech Republic). *Xenopus* uptake assays were carried out as follows: oocytes were preincubated in Kulori pH5 for 5 min, transferred to Kulori pH 5 with substrate for 60 min incubation, followed by four washes and transferred to Eppendorf tubes (one oocyte per tube or five oocytes). Excess washing buffer was removed and oocytes were busted in 50 μl of 50% MeOH and the homogenate was left in the freezer for 2 h. This was followed by centrifugation at 20,000*g* for 15 min to pellet remaining proteins. The supernatant was transferred to new tubes and diluted with 60 μl H_2_O. The diluted samples were then filtered through a 0.45 μm PVDF-based filter plate (MSHVN4550, Merck Millipore) and subsequently analysed by analytical LC-MS. Extracted data were analysed using Microsoft Excel, statistical analysis and data plotting were done using SigmaPlot version 13.0 (Systat software, USA).

### Analysis of hormones and hormone-analogs by LC-MS

Compounds in extracts were directly analysed by LC-MS/MS. Chromatography was performed on an Advance UHPLC system (Bruker, Bremen, Germany). Separation was achieved on a Kinetex 1.7 u XB-C18 column (100 × 2.1 mm, 1.7 μm, 100 Å, Phenomenex, Torrance, CA, USA). Formic acid (0.05%) in water and acetonitrile (supplied with 0.05% formic acid) were employed as mobile phases A and B, respectively. The elution profile was: 0–0.2 min, 2% B; 0.2–0.9 min, 2–30% B; 0.9–3.2 min 30–100% B, 3.2–3.7 min 100%; 3.7.–3.8 min 100–2% B and 3.8–5.0 min 2% B. The mobile phase flow rate was 400 μl min^−1^. The column temperature was maintained at 40 °C. The liquid chromatography was coupled to an EVOQ Elite TripleQuad mass spectrometer (Bruker) equipped with an electrospray ion source operated in combined positive and negative ionization mode. The instrument parameters were optimized by infusion experiments with pure standards. The ion spray voltage was maintained at −4,000 V for GA and Sinigrin and +4,000 V GA-fluorescein analysis, respectively. Cone temperature was set to 300 °C and cone gas to 20 p.s.i. Heated probe temperature was set to 200 °C and probe gas flow to 50 p.s.i. Nebulizing gas was set to 60 p.s.i. and collision gas to 1.6 mTorr. Nitrogen was used as probe and nebulizing gas and argon as collision gas. Active exhaust was constantly on. Multiple reaction monitoring (MRM) was used to monitor analyte parent ion→product ion transitions: MRM transitions were chosen based on direct infusion experiments. Detailed values for mass transitions can be found in Supplementary Table 4. Both Q1 and Q3 quadrupoles were maintained at unit resolution. Bruker MS Workstation software (Version 8.2, Bruker) was used for data acquisition and processing. Linearity in ionization efficiencies were verified by analysing dilution series of standard mixtures. Sinigrin was used as internal standard but not used for quantification. Quantification of all compounds was achieved by external standard curves diluted with the same matrix as the actual samples. Identification of all analytes was achieved by their specific MRM transitions and by chromatographic separation (Supplementary Figs 7, 8 and Supplementary Table 4).

For data presented in Fig. 1b,c and Supplementary Fig. 1a chromatography was performed on an Agilent 1,100 Series LC (Agilent Technologies, Germany). Separation was achieved on a Zorbax SB-C18 column (Agilent; 1.8 μm, 2.1 × 50 mm) at a flow rate of 0.2 ml min^−1^. Formic acid (0.1%) in water and acetonitrile (supplied with 0.1% formic acid) were employed as mobile phases A and B, respectively. The elution profile was: 0–0.5 min, isocratic 6% B; 0.5–12.5 min, linear gradient 6–55% B; 12.5–13.1 min, linear gradient 55–95% B; 13.1–15.5 isocratic 95% B; 15.60–20 min, isocratic 6% B. The flow rate was increased to 0.3 ml min^−1^ in the interval 15.2–17.5 min. The column temperature was maintained at 35 °C. The Bruker HCT-Ultra ion trap mass spectrometer (Bruker Daltonics) was run in positive electrospray mode. Bruker MS Workstation software (Bruker) was used for data acquisition and processing. Linearity in ionization efficiencies were verified by analysing dilution series of standard mixtures. Quantification of all compounds was achieved by external standard curves diluted with the same matrix as the actual samples.

### Predicted topology of NPF3

Topologies were defined by the online HMM-top tool (http://www.enzim.hu/hmmtop/) and visualized by the TMRPres2D software (http://biophysics.biol.uoa.gr/TMRPres2D/download.jsp).

### Phylogenetic tree of the *Arabidopsis* NRT/PTR family

Protein sequences for *Arabidopsis thaliana* NRT/PTR family members were retrieved from TAIR (https://www.arabidopsis.org). Phylogenetic relationship was defined using (http://www.phylogeny.fr/)^59^,^60^,^61^,^62^ and visualized with FigTree software (http://tree.bio.ed.ac.uk/software/figtree/).

## Additional information

**How to cite this article:** Tal, I. *et al*. The *Arabidopsis* NPF3 protein is a GA transporter. *Nat. Commun.* 7:11486 doi: 10.1038/ncomms11486 (2016).

## Supplementary Material

Supplementary Information Supplementary Figures 1-8, Supplementary Tables 1-4 and Supplementary References

Supplementary Data 1 Collection of T-DNA insertion lines screened for root endodermis deficient GA-Fl accumulation
