# Supplementary material for: The Arabidopsis NPF3 protein is a GA transporter
Source: Nat Commun. 2016 May 3;7:11486. doi: 10.1038/ncomms11486 (PMC4857387; doi:10.1038/ncomms11486)
Supplement: Supplementary Information — Supplementary Figures 1-8, Supplementary Tables 1-4 and Supplementary References [file ncomms11486-s1.pdf]

SUPPLEMENTARY INFORMATION

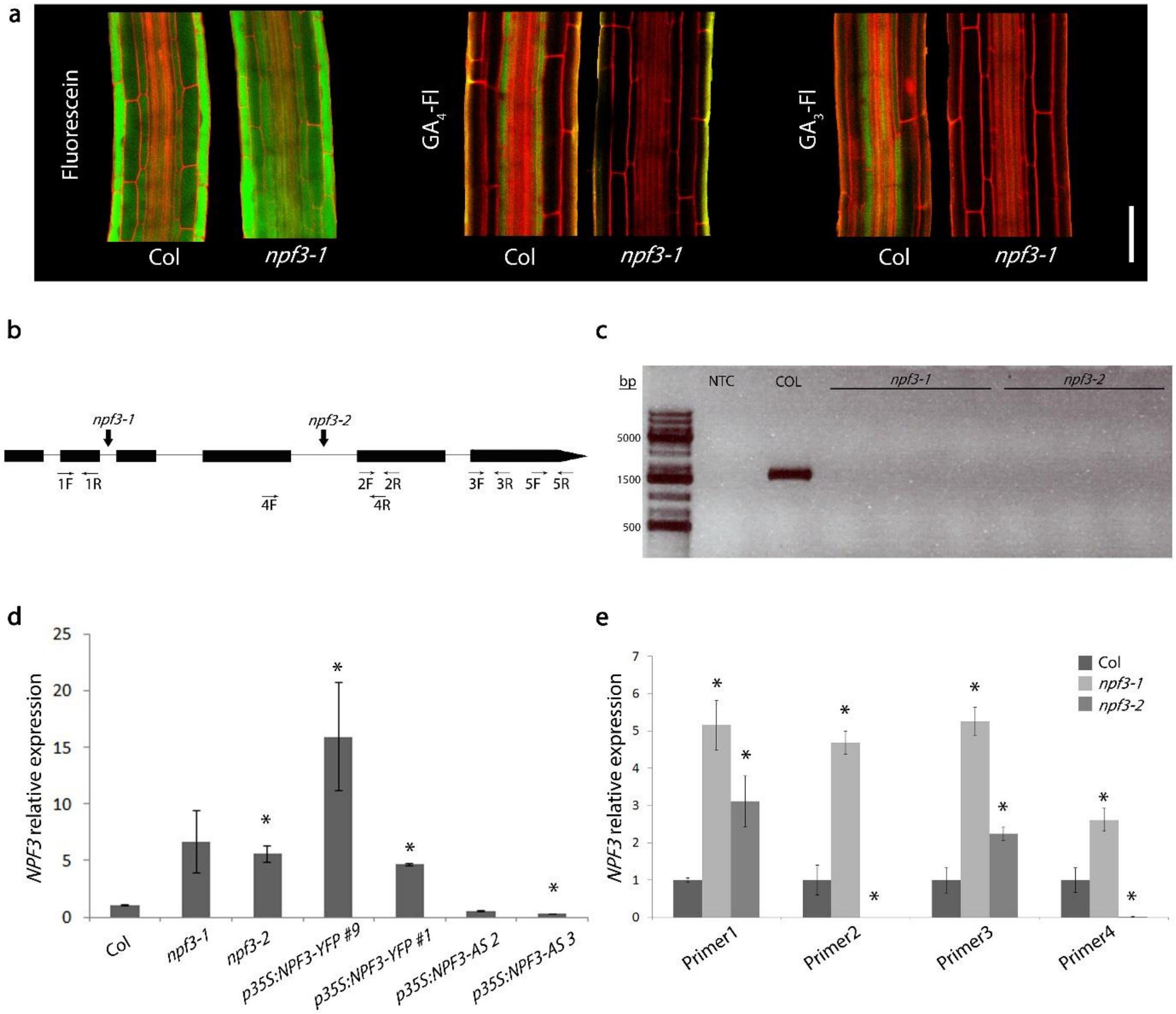

Supplementary Fig 1. *npf3* loss-of-function molecular characterization.

a) Distribution of fluorescein (Fl) and fluorescently tagged GAs, GA_3-Fl or GA_4-Fl in elongating endodermal cells of roots treated with the fluorescent molecules for 3h in the indicated genotypes. Fl concentration 1 µM. GA-Fl concentration 5 µM. Bar = 50 µm **b)** *NPF3* (AT1G68570) gene model. Vertical arrows indicate the T-DNA insertions in *npf3-1* (SALK_130095) and *npf3-2* (GK-356G08). Horizontal arrows indicate the primers used for PCR and qPCR amplification. **c)** Accumulation of full length *NPF3* cDNA using primers 1F and 3R as indicated in Fig. b above. **d)** Relative *NPF3* expression in *npf3* loss of function and overexpression lines quantified by qRT–PCR with primer set 5 as indicated in Fig. b above. **e)** Relative *NPF3* expression in *npf3* loss-of-function mutants quantified by qRT–PCR with primer sets 1, 2, 3, 4 as indicated in panel b. Relative gene expression (for d-e) was calculated by normalizing to the value of the WT, reference gene is *PP2A*. Values are the mean ± SE of at least three biological replicates. * Significantly different relative to Col at $P \leq 0.05$ by Student *t-test*.

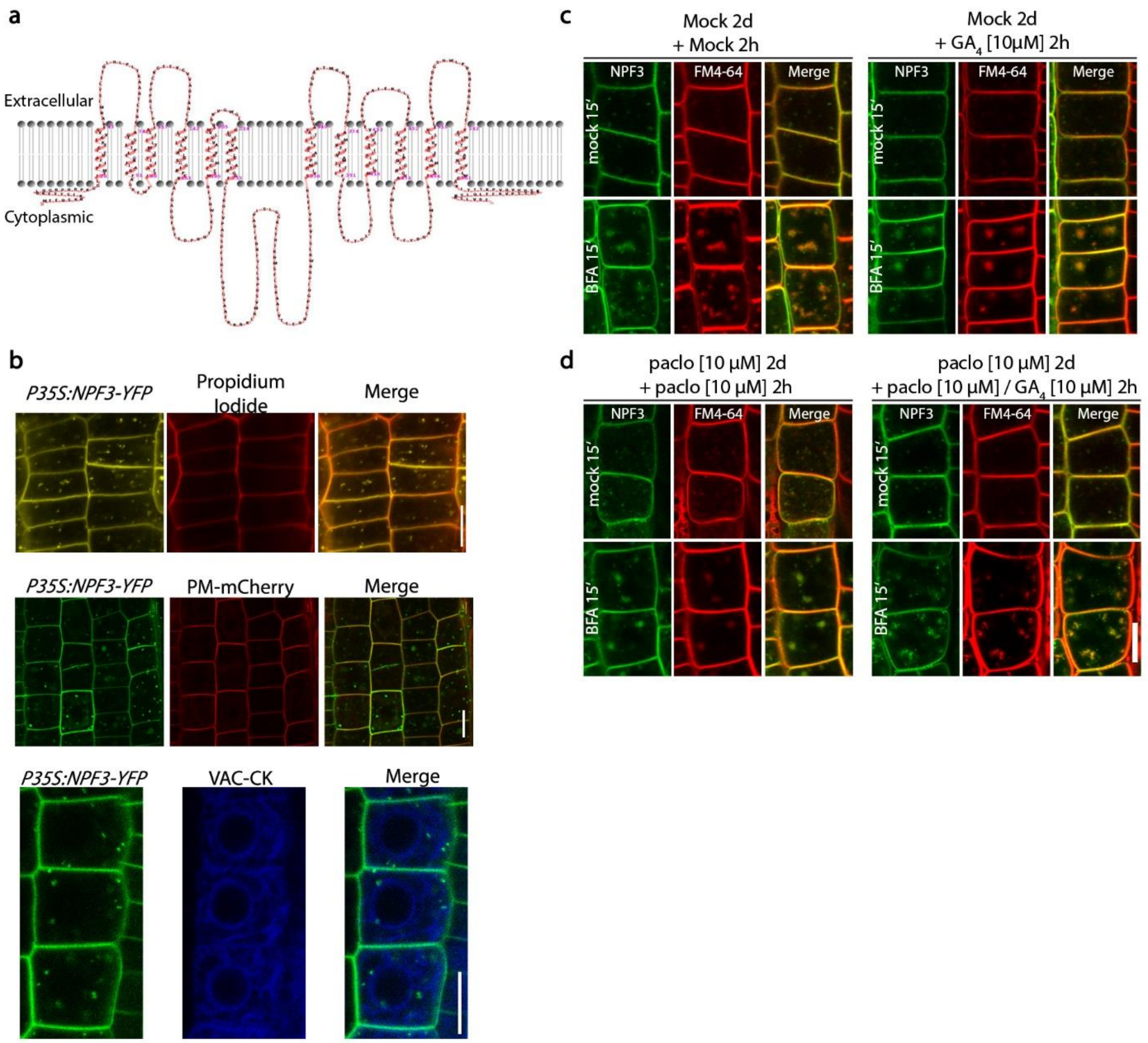

Supplementary Fig 2. GA_4 does not affect NPF3 localization or its BFA sensitivity.

a) Predicted topology of NPF3**. b)** Root tip confocal microscopy images of NPF3-YFP localization counterstained with the indicated markers: propidium iodide, VAC-CK (VAC-CFP, tonoplast marker[1]), PM-mCherry (plasma membrane marker [1]). Scale bar = 10 µm. **c-d)** Confocal microscopy images of *p35S:NPF3-YFP* root epidermal cells in response to BFA treatment (50 µM, 15 min). Seedlings were grown on control (mock) growth media (c) or 10 µM paclo (d) and treated with GA_4 (10 µM 2h) (right panels) or without (left panels). (*n*=6). Scale bar = 10 µm.

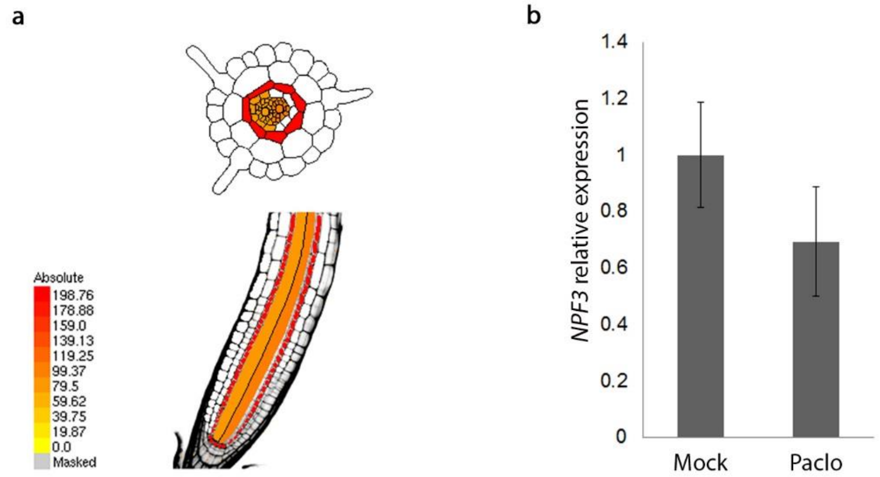

Supplementary Fig 3. *NPF3* transcript is translated in the root endodermis.
a) *NPF3* translation levels derived from root cell-type-specific protoplasts *Mustroph et al., 2009* [2]. **b)** *NPF3* expression levels in paclo grown (10 µM, 3.5 h) Col seedlings. Quantified by qRT–PCR with *PP2A* as a reference gene**.** Values are the mean ± SE of three biological replicates. The paclo treatment is not significantly different relative to Col at $P \leq 0.01$ by Student *t-test*.

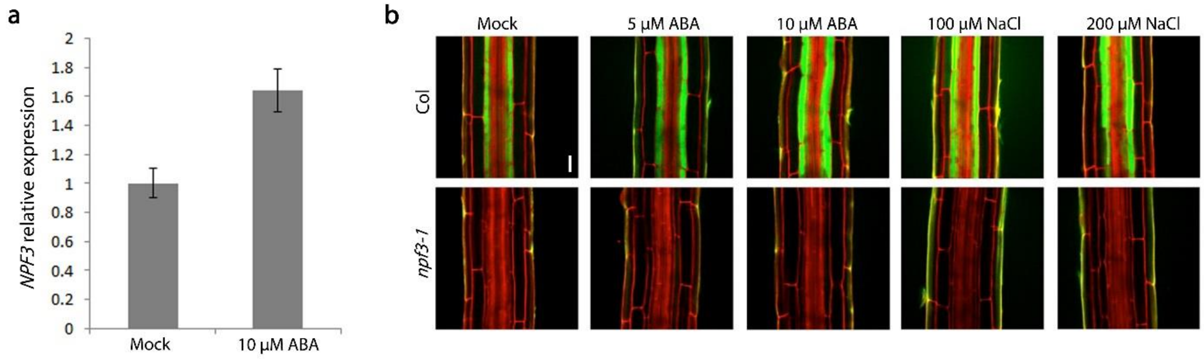

Supplementary Fig 4. ABA induces *NPF3* transcription as well as an increases GA_3-Fl uptake in the root.
a) Relative *NPF3* expression in response to ABA treatment (10 µM, 3h) quantified by qRT–PCR with *PP2A* as a reference gene. Values are mean ± SE of 4 biological replicates. **b)** Distribution of GA_3-Fl (5 µM, 3h) in the root elongation zone for Col (WT) and *npf3-1* mutant in the absence or presence of ABA and NaCl at the indicated concentrations. Scale bar = 10 µm.

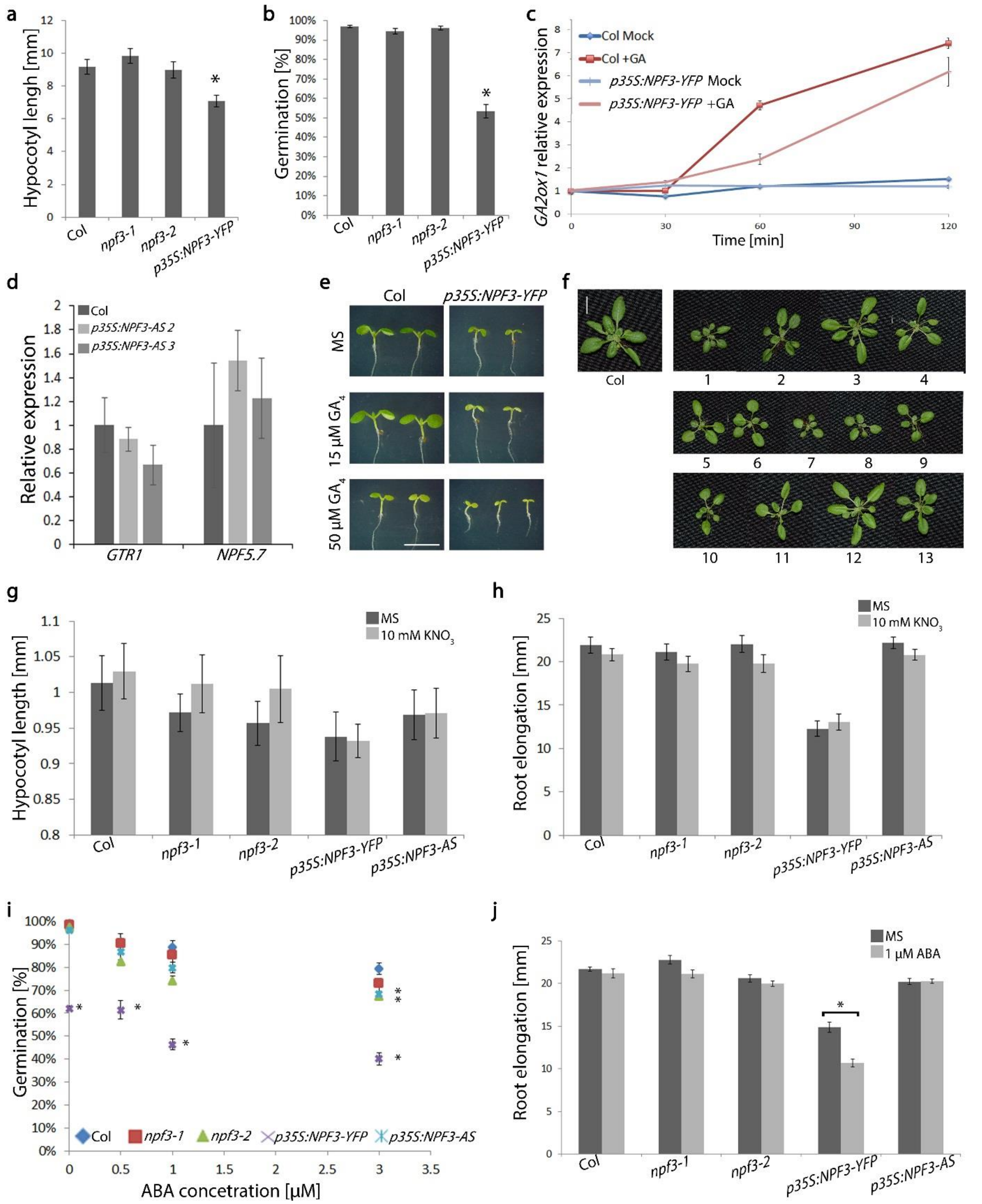

Supplementary Fig 5. *NPF3* loss and gain-of-function phenotypic characterization.

a) Hypocotyl length of etiolated 3 day old seedlings. Values are means ± SE *n≥15*. **b)** Germination rate determined as seed coat rupture after 2 days. Values are means ± SE *n=348* divided into four biological replicates. **c)** *GA2ox1* expression levels in paclo grown seedlings (6 days, 5 μM) in response to GA_4 treatment (10 μM) at the indicated times points, quantified by qRT–PCR with *Actin8* as a reference gene.

d) *GTR1* and *NPF5.7* expression levels in 10 day-old *p35S:NPF3-Antisense* seedlings, quantified by qRT–PCR with *PP2A* as a reference gene. **e)** Representative images of WT and *p35S:NPF3-YFP* 7 day-old-seedlings treated with increasing amounts of GA_4. Bar = 0.5 cm **f)** Phenotype of 3 weeks old *p35S:NPF3-YFP* lines grown on soil. Scale bar = 1 cm. **g-h)** Effect of nitrate on hypocotyl (g) and root (h) elongation. Seeds germinated and grown on MS or 10 mM KNO_3. Elongation measured on day 8. Values are means ± SE $n \geq 13$. **i)** Germination rate on increasing ABA concentrations determined as seed coat rupture after 2 days. Values are means ± SE $n=200$ divided into three biological replicates for each genotype. **j)** ABA effect on root elongation of indicated lines. Seedlings were transferred to 1 µM ABA plates at day 4, root elongation measured after 4 additional days. Values are means ± SE $n \geq 10$.

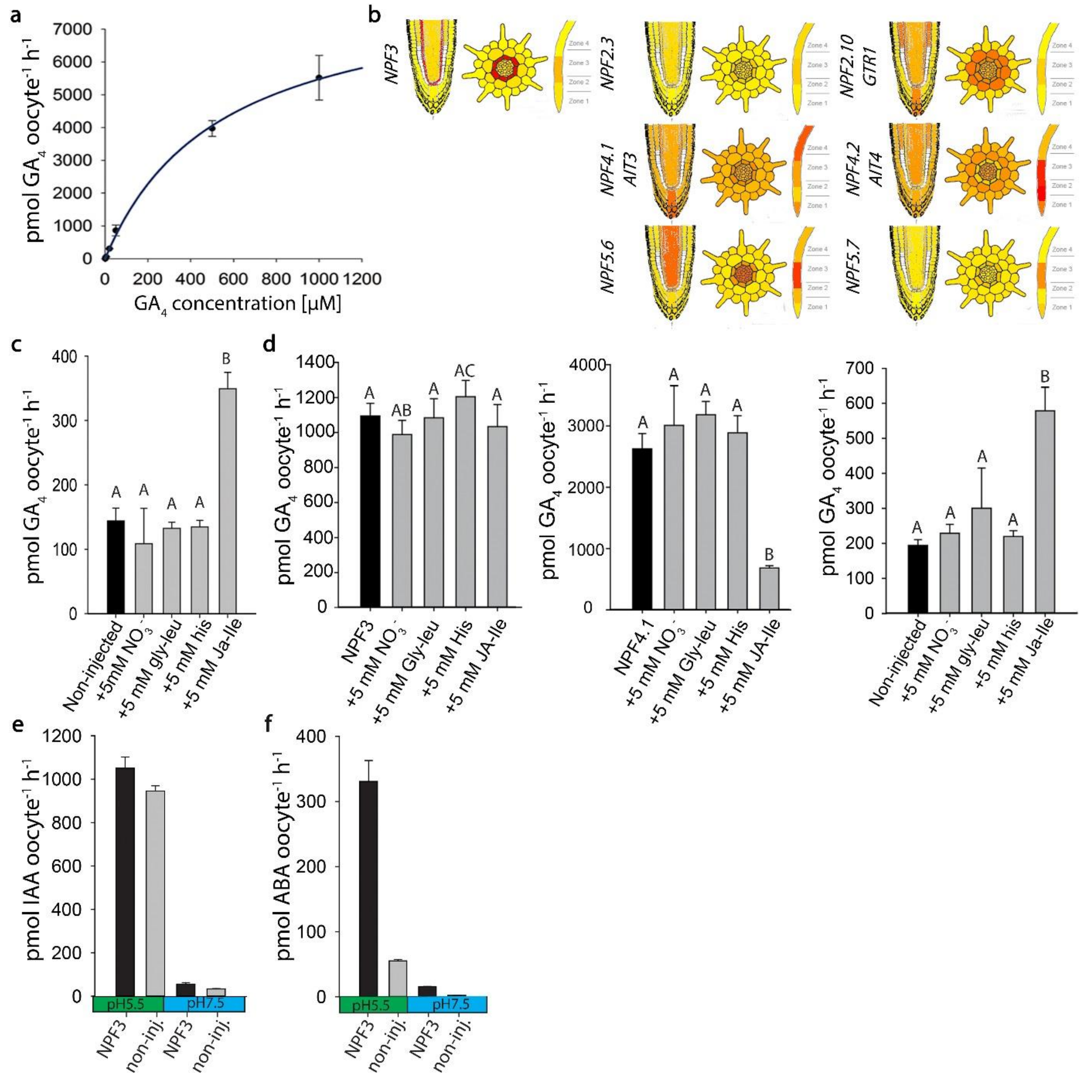

Supplementary Fig 6. NPF3 is a GA transporter in *X. laevis o*ocytes.

a) GA_4 uptake in oocytes exposed to increasing concentrations GA_4 at pH 5.5. Error bars are SD, *n =3x5* oocytes. **b)** Expression levels of the indicated NPFs derived from root cell-type-specific protoplasts *Iyer-Pascuzzi et al., 2011* [3]. **c)** Competition of GA_4 diffusion in non-injected oocytes. Non-injected oocytes were exposed to 300 µM GA_4 alone or 300 µM GA_4 together with either 5 mM NO_3^-, 5 mM dipeptide (Gly-leu), 5 mM histidine (His) or 5 mM JA-isoleucine (JA-Ile). Error bars are SD, n = 5. **d)** Independent repetition of competition assays in Fig. 4g. Competition of GA_4 uptake mediated by NPF3 and NPF4.1 (AIT3) expressing oocytes. Oocytes expressing either NPF3 or NPF4.1 were exposed to 300 µM GA_4 alone or 300 µM GA_4 together with either 5 mM NO_3^-, 5mM dipeptide (Gly-leu), 5 mM histidine (His) or 5 mM Ja-isoleucine (JA-Ile). Error bars are SD, n = 5. Groups are determined by one-way ANOVA and ($P < 0.05$). non-inj., non-injected. **e-f)** Uptake of IAA (e) and ABA (f) at pH 5.5 and pH7.5. 15 NPF3 expressing or non-injected oocytes were incubated for 1 h in 100 µM ABA or IAA at pH5.5 or pH7.5. Error bars are SD, n = 3.

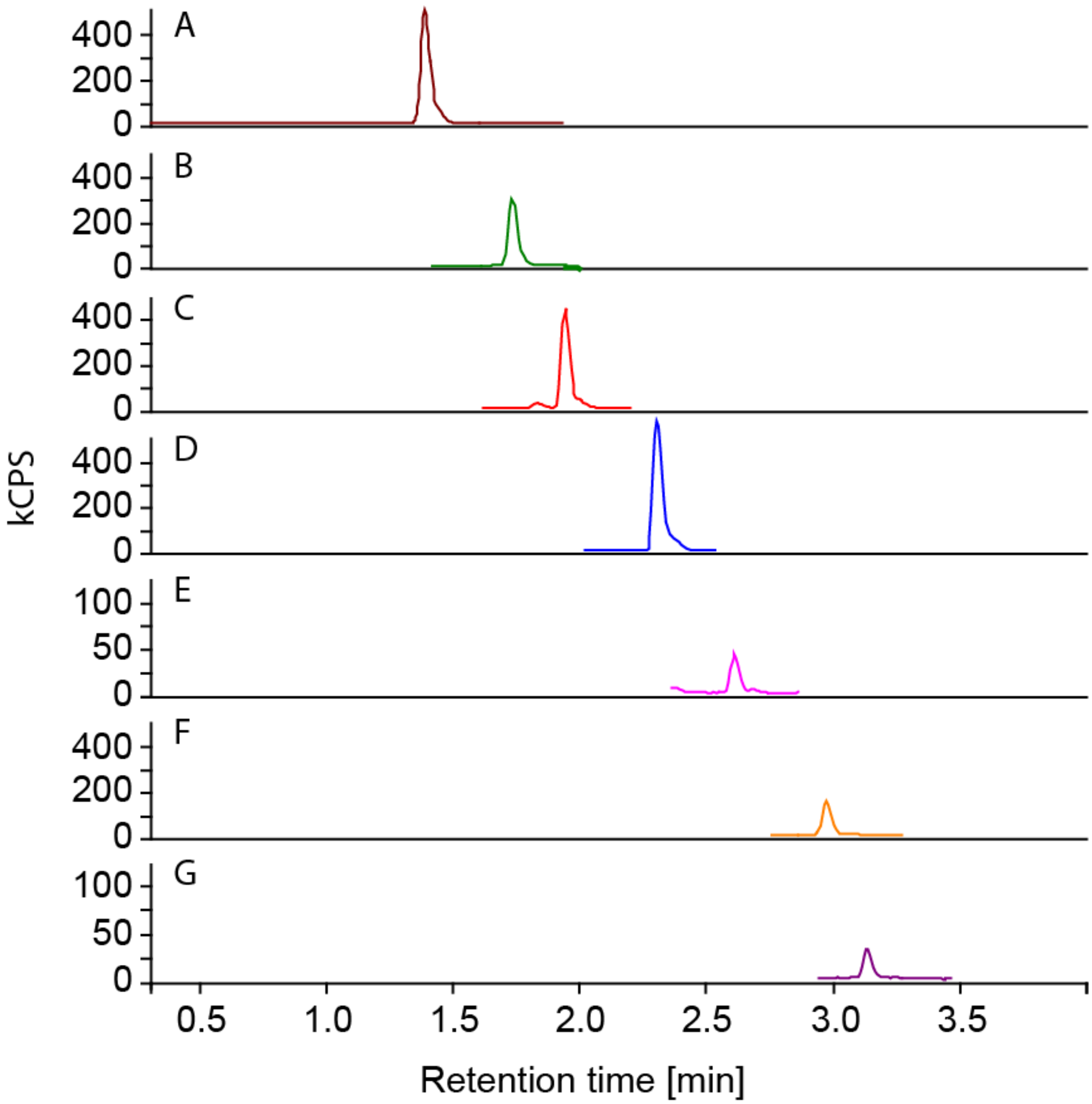

Supplementary Fig 7. LC/MS traces of all analyzed GAs.

Shown are the traces for the quantifier parent ion → daughter ion transitions (for details see also Supplemental Table S2) for all GAs individually in order of their retention time. A) Sinigrin (IS), B) GA_8, C) GA_3, D) GA_{20}, E) GA_4, F) GA_9, G) GA_{12}.

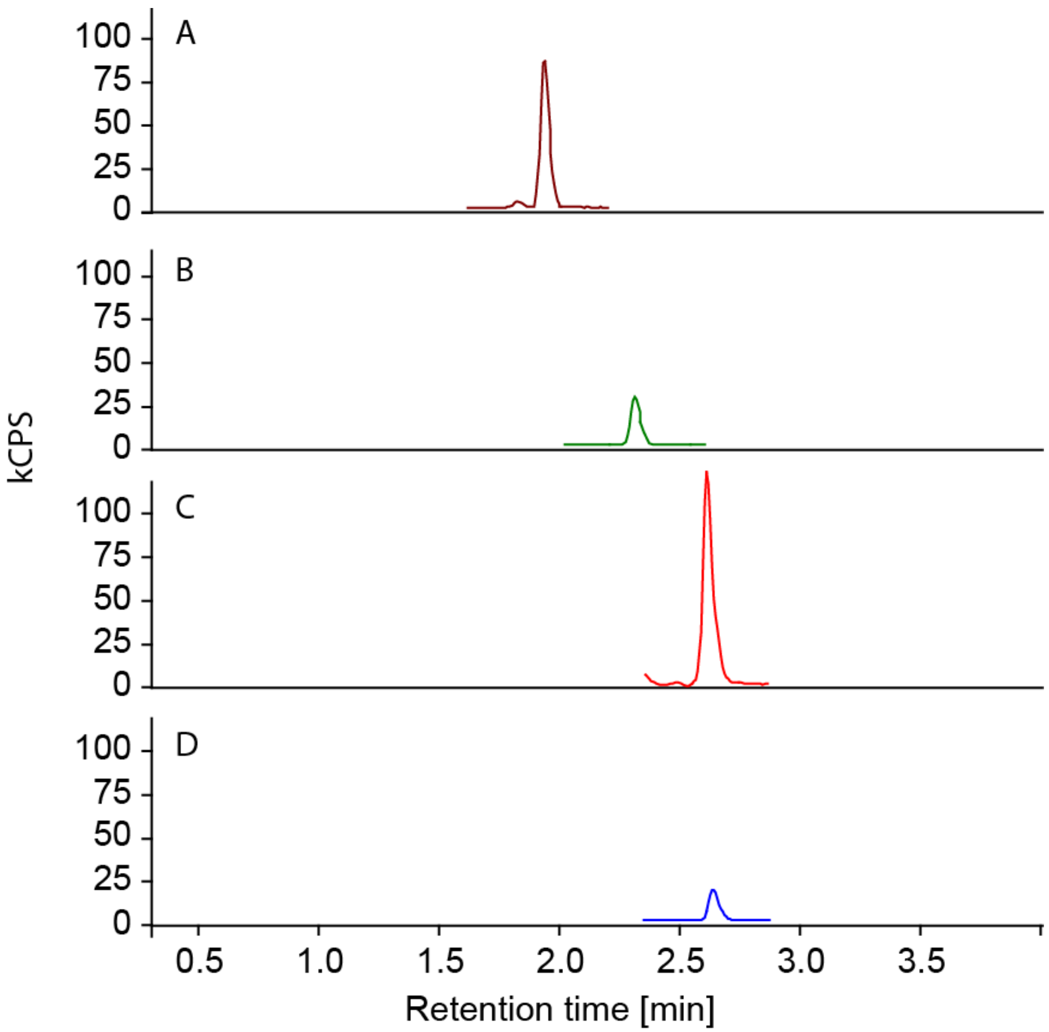

Supplementary Fig 8. LC/MS traces of GA_3 and GA_4 and their derivatives coupled to fluorescein.
Shown are the traces for the quantifier parent ion → daughter ion transitions (for details see also Supplemental Table S4) for all GAs individually in order of their retention time. A) GA_3, B) GA_3-FL, C) GA_4, D) GA_4-FL.

Supplementary table 1: primers for T-DNA lines genotyping

Primer name	Primer sequence
npf3-1 F (SALK_130095)	GCAGCCAACACTCTCACAAA
npf3-1 R (SALK_130095)	CTGATCAGCACCAAAAGCAA
npf3-2 F (GK-356G08)	CAACTGGGTGAAACCAGACA
npf3-2 R (GK-356G08)	TTACTATTGCCGCCTTGTCC
Lbb1.3 (SALK)	ATTTTGCCGATTTCGGAAC
KN44 (GK)	ATAATAACGCTGCGGACATCTACATTTT

Supplementary table 2: primers for cloning *NPF3* overexpression and reporter lines

Construct	Primer sequence	Source	Destination Gateway vector
p35S:NPF3-antisense	For: caccATGGAGGAGCAAAGCAAGAA<br>Rev: TCATTCATCAACTAAACTCCTATTTGACA	cDNA (Col-0)	pH2GW7
p35S:NPF3-YFP	For: caccATGGAGGAGCAAAGCAAGAA<br>Rev: TTCATCAACTAAACTCCTATTTGACA	DNA (Col-0)	pH7YWG2
pNPF3:LUC	For: caccCGACCAATCATGGGAAGAAG<br>Rev: GATGTTTCTGATTGTTTCTTGTAATAA	DNA (Col-0)	pFlash

Supplementary table 3: primers for Quantitative RT–PCR

PP2A For	TAACGTGGCCAAAATGATGC
PP2A Rev	GTTCTCCACAACCGCTTGGT
NPF3 primer set 1 For	ATATTCAGCGAACGAGATA
NPF3 Primer set 1 Rev	ACCAAGGAGAGGAGTAAGAC
NPF3 primer set 2 For	ATAGTAACGGAGGAAGACAA
NPF3 Primer set 2 Rev	ATAGGCTGTGATTAGGAGAA
NPF3 primer set 3 For	CTGGTGACTTTGGTTCATA
NPF3 Primer set 3 Rev	GGACTACTATCTTCCTTGCT
NPF3 primer set 4 For	ACTCACCCACACCAAACACA
NPF3 Primer set 4 Rev	ATTCGGATGACGGATTTGAG
NPF3 primer set 5 For	ATGTGAGCACTTTGCTGGTG
NPF3 Primer set 5 Rev	TCTTGGCGCACCATAGATAA
Actin 8 For	TCAGCACTTTCCAGCAGATG
Actin 8 Rev	CTGTGGACAATGCCTGGAC

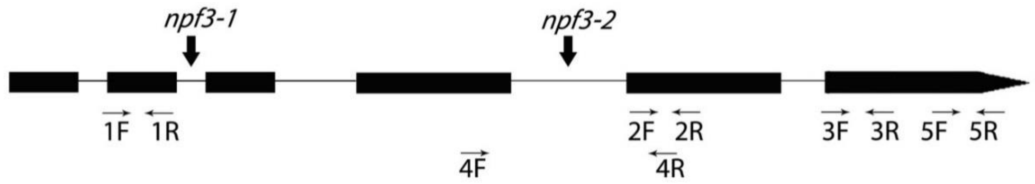

NPF3 (AT1G68570) gene model (enlarged from Sup 1b). Vertical arrows indicate the T-DNA insertions causing the non-functional alleles selected from the screens *npf3-1* (SALK_130095) and *npf3-2* (GK-356G08). Horizontal arrows indicate the primers used for PCR and qPCR amplification.

Supplementary Table 4: MRMs for gibberellic acid and fluorescein-labelled GA by LC/MS/MS

Compound	RT	Q1	Q3	CE [eV]
GA_3	1.94	345.2	243.1^{Q}	25
		345.2	239.1	12
		345.2	221.1	21
GA_3-fluorescien	2.28	835.3	773.2^{Q}	-27
		835.3	507.1	-41
		835.3	463.9	-50
GA_4	2.63	331.1	243.0^{Q}	16
		331.1	225.1	15
		331.1	213.0	30
GA_4-fluorescien	2.55	821.2	507.0^{Q}	-46
		821.2	253.0	-41
		821.2	463.9	-58
GA_8	1.73	363.2	275.1^{Q}	15
		363.2	257.1	15
		363.2	119.2	23
GA_9	3.00	315.2	271.1^{Q}	16
		315.2	253.1	23
GA_{12}	3.13	331.2	313.1^{Q}	26
		331.2	269.1	33
		331.2	287.0	21
GA_{20}	2.30	331.2	287.0^{Q}	19
		331.2	313.1	26
		331.2	269.1	20
Sinigrin (IS)	1.38	358.0	75.0	30
		358.0	97.0^{Q}	22
		358.0	259.0	20

[Q]Quantifier ion used for quantification of the respective compound. Other transitions were used for compound identification together with retention times compared to those of standards. IS = internal standard.

Bibliography

1. Nelson BK, Cai X, Nebenführ A. A multicolored set of in vivo organelle markers for co-localization studies in Arabidopsis and other plants. *The Plant Journal* **51**, 1126-1136 (2007).

2. Mustroph A*, et al.* Profiling translatomes of discrete cell populations resolves altered cellular priorities during hypoxia in Arabidopsis. *Proc Natl Acad Sci U S A* **106**, 18843-18848 (2009).

3. Iyer-Pascuzzi AS*, et al.* Cell identity regulators link development and stress responses in the Arabidopsis root. *Developmental cell* **21**, 770-782 (2011).
